# Effect of Weightlessness on the 3D Structure Formation and Physiologic Function of Human Cancer Cells

**DOI:** 10.1155/2019/4894083

**Published:** 2019-04-03

**Authors:** Zheng-Yang Chen, Song Guo, Bin-Bin Li, Nan Jiang, Ao Li, Hong-Feng Yan, He-Ming Yang, Jin-Lian Zhou, Cheng-Lin Li, Yan Cui

**Affiliations:** ^1^Department of General Surgery, 306 Hospital of PLA, Beijing 100101, China; ^2^Department of General Surgery, 306 Hospital of PLA-Peking University Teaching Hospital, Beijing 100101, China; ^3^Department of General Surgery, PLA 306 Clinical Hospital of Anhui Medical University, Beijing 230000, China; ^4^Department of Pathology, 306 Hospital of PLA, Beijing 100101, China

## Abstract

With the rapid development of modern medical technology and the deterioration of living environments, cancer, the most important disease that threatens human health, has attracted increasing concerns. Although remarkable achievements have been made in tumor research during the past several decades, a series of problems such as tumor metastasis and drug resistance still need to be solved. Recently, relevant physiological changes during space exploration have attracted much attention. Thus, space exploration might provide some inspiration for cancer research. Using on ground different methods in order to simulate microgravity, structure and function of cancer cells undergo many unique changes, such as cell aggregation to form 3D spheroids, cell-cycle inhibition, and changes in migration ability and apoptosis. Although numerous better experiments have been conducted on this subject, the results are not consistent. The reason might be that different methods for simulation have been used, including clinostats, random positioning machine (RPM) and rotating wall vessel (RWV) and so on. Therefore, we review the relevant research and try to explain novel mechanisms underlying tumor cell changes under weightlessness.

## 1. Introduction

With the great strides of the space industry, people are staying in space for increasing amounts of time. Long flights in space can cause severe effects on human physiology and health [1, 2]. Now, a great number of evidence have suggested that many human health problems during long space flights may be due to alterations of the expression of genes and proteins induced by SMG. It is confirmed that more than 1600 genes expression have been altered when cells are exposed to SMG [3]. These alterations in genes result in corresponding alterations in the cytoskeleton—(microtubule [MT], microfilament [MF] and intermediate filament [IF]), ECM, growth pattern, migration abilities, cell cycle, proliferation, and apoptosis. Recently, researchers have shown that simulated microgravity (SMG) can induce these alterations not only in normal cells [4–6], but also in tumor cells [7–9].

Malignant tumors are still the main cause threatening human life and health. With the developments in molecular biology, cell biology, immunology and other related disciplines, treatment methods involving induction of differentiation and apoptosis have emerged, which have allowed breakthroughs in the treatment of some tumors. However, cancer mortality remains high due to limitations in diagnosis, treatment (e.g., surgery, chemotherapy, radiation therapy), and care. Therefore, it is necessary to find a new way to overcome these difficulties. Compared to normal gravity (NG), the morphological function of cancer cells is obviously altered as a result of the unique microgravity in space, which provides a new method to study these problems. However, real microgravity is rarely achieved by orbital laboratories [10]. Therefore, SMG is simulated by using the so called Random Positioning Machine (RPM), “clinostat” and others; these devices can produce many of the physical effects of microgravity (*μ*g) by providing a vector averaged reduction of apparent gravity [11–15]. However, compared to the one axis clinostat, the amount of shear forces in two axis RPM modes is higher [16]. The highest shear forces on the RPM are found along the flask walls; they can reach up to a few 100 mPa (1 dyn/cm^2^) depending on the rotational velocity, whereas in the “abulk volume” they are always smaller and usually about 10 mPa (0.1 dyn/cm^2^) [17]. These shear forces have a strong influence on cell metabolism and function in general. For instance, doubling the fluid shear force caused by the simulated microgravity increased glucose metabolism and reduced cell aggregation in BHK cells [18]. This shows that higher shear forces are acting on the cancer cells during the RPM [16], which might lead to the different results on the two axis RPM and one axis clinostat.

The adverse effects of various external factors lead to the body fluids distribution, often accompanied by cell dysfunction and cytoskeletal changes. Cells form complex three-dimensional (3D) spherical structures of different diameters under these conditions [20, 39, 46, 49, 52]. Cell cycle changes were not consistent under simulated microgravity; for instance, the G_2_/M was increased or decreased in MCF-7 and SGC-7901 cells using a MG-6C and MG-3 clinostat system [42, 44]. Apoptosis induced by apoptosis-related protein was increased in different cancer types under SMG using a 2D clinostat and RPM in ML-1, U251 and MDA-MB-231 cells [25, 41, 45]. However, apoptosis was reduced or no obvious apoptosis was seen in other cancer cell lines such as MIP-101 and A549 under SMG using a 2D clinostat and RPM [19, 24, 53]. The reasons for these two contradictory results in apoptosis are still not clear. The former may be related to the increasing apoptosis-related protein [25, 41, 45], while the later may be related to lower expression of EGF-R, TGF, and cell proliferation markers [19, 24, 53]. It is clear that there are differences due to the rotation devices (e.g., clinostat system and RPM) that might account for the alteration in cell cycle, apoptosis and proliferation. 2D monolayers could be formed under normal conditions, which were arranged regularly. However, under SMG using a RCCS, cells were inclined to gather and form aggregates in a 3D environment, interacting on multiple levels [21, 54]. The changes in cell morphology and motility observed under SMG using a 2-D clinostat system might lead to morphological disorders during development [42].

As shown in Table 1, although many experiments on the effects of real and simulated microgravity on human cancer cells had been carried out over several years, the biological effects of microgravity on 3D spheroid and morphological functions of human cancer cells still need to be explored. In this review, we summarize the main effects of microgravity on 3D structure formation and the morphological functions of human cancer cells and propose some novel views on this basis.

## 2. Ground-Based Facilities for Simulation of Microgravity

Recent years, different kinds of ground-based facilities (GBFs) have been designed aiming to simulate microgravity on ground [55]. The SMG should be used only for experiments conducted in GBFs where the direction of the gravity vector has undergone a constant (2-D clinostat) or random (RPM) change, with the gravity level averaged to near zero along with rotation and time [55]. In a sense, the microgravity analog of SMG created by GBFs has been regarded as “functionally near weightlessness” and was seldom equal to the “*μ*g” in space or “weightlessness” [56]. Here, several frequently used GBFs, including clinostats, RPM and RWV, are described.

### 2.1. Clinostats

Aiming to construct a machine to simulate microgravity, the clinostats were developed. Clinostats with one axe are called 2-D clinostats, while those with two axes are called 3-D clinostats and will be described in the RPM (Section 2.2). The choosing of rotational axes, speed, and direction of rotation were designed according to different organisms and experimental requirements. 2-D clinostats rotate perpendicularly to the direction of the gravity vector, while the one rotation axis represents a classical and well-established model to simulate SMG [56]. Although several studies have shown that the results using 2-D clinostats were similar to those found under real microgravity conditions, we can not neglect its shortcoming [57, 58]. If the velocity is too high, the clinostat turns into a centrifuge. Furthermore, just cells small enough to fit centered into the rotation axis can be rotated with the clinostat, because any part of a sample larger than the rotation axis would experience centrifugal forces [59], not only this situation in the clinostats, but also in the RPM under larger samples.

### 2.2. Random Positioning Machine (RPM) – 3-D Clinostats

Clinostats with two axes are called 3-D clinostats. Biological samples in 3-D clinostats are rotated along two independent axes to change their orientations at constant speeds and directions relative to the gravity vector, thereby eliminating the effect of gravity [56, 60]. Specifically, RPMs refer in particular to these 3-D clinostats that rotate with random changing speed and direction relative to the gravity vector [61]. Compared with 2-D clinostats, 3-D clinostats were supposed to be capable of increasing the quality of simulations, especially for large organisms.

### 2.3. Rotating Wall Vessel (RWV)

The RWVs were first designed for cell cultures by the National Aeronautics and Space Administration (NASA) Johnson Space Center (Houston, TX, USA). The RWV is a vessel that can rotate horizontally with no internal mechanical agitator and thus can generate low-shear modeled microgravity (LSMMG) [62]. The bottom of vessel is designed a silicone membrane which can deliver oxygen via diffusion and avoid the production of bubbles. The hollow disk or cylinder of RWV rotates perpendicularly to the direction of the gravity vector with one rotational axis. The hollow disk or cylinder provides a culture environment that is characterized by low shear and low turbulence as a result of the cells suspended under specific culture conditions. Consequently, cells in the vessel are in a state of free fall, but it is impossible for them to reach the bottom of the vessel owing to the constant rotation of the RWV. Unlike clinostats and RWV, cells in RWV are grown on microcarrier beads to provide a solid support and the cells readily attach and cover the surface of the microcarriers. Other RWVs, like rotating cell culture systems (RCCSs) and high-aspect rotating vessels (HARVs), were originated from RWVs basing on similar physical principles using different configurations and have also been applied to study the effects of microgravity [63].

## 3. Different Cell Morphologies in Microgravity Environment

Cells always maintain their own normal shape through their inner structure and the tension on their own surface, as well as through mechanical stress. Because the cytoskeletal was affected by simulated microgravity [64], some cell components are rearranged in order to disperse the tension and pressure, resulting in cells to be more rounded. Therefore, changes in mechanical stress can affect the growth and biochemical properties of the cell through a force balance in the cell and its cytoskeleton; i.e., when the cell is directly subjected to mechanical stimulation* in vitro*, its morphology and function will change. It has been found that cytoskeletal rearrangement can induce cell morphological changes [65, 66]. The cytoskeleton transmits and distributes the molecular distortion force on mechanical receptors through direct contact with the molecules on the cell membrane, and the mechanical signal is finally displayed on the effect point through the distortion force of the effector molecules [67]. Once mechanical stress is perceived, PKC and MAPKs are activated, resulting in* c-fos*, and* c-jun* expression [68, 69]. In addition, mechanical stress can directly activate the tyrosine kinase pathway and transmit mechanical signals to guanine nucleoside conjugate protein, and also directly activate MAPK and RAF-1 kinase [70]. Real and simulated microgravity activates these signal pathways, resulting in adaptive changes and abnormal expression of various adhesion molecules, which finally leads to altered 3D structure formation and morphological functions (e.g., cytoskeleton, ECM, cycle, migration, proliferation and apoptosis) of cells, especially cancer cells.

### 3.1. 3D Spheroid Structure Formation

Cells form a 2D monolayer structure in the medium under normal conditions. The structure surface is smooth and regular without complex structure formation and with limited interaction. However, in the microgravity environment, the adverse effects of various factors acting on cells lead to the body fluids distribution, along with cell dysfunction, cytoskeletal changes, and genetic abnormalities. These cancer cells form complex 3D spheroid structures in real and simulated microgravity [20, 25, 27, 28, 31, 34–36, 39, 46, 48]. Studies have found that these 3D spheroids range from 0.3 mm to 0.5 mm in diameter [20, 25, 36, 39, 46]. The reason why the diameter of 3D spheroids is maintained at 0.3-0.5 mm is probably because cells in the sphere might be in a relatively anoxic environment and induce high expression of hypoxia-inducible factor-1 (HIF-1), which acts on the cytoskeleton, reduces proliferation, and induces apoptosis; on the other hand, abnormal expression of adhesion molecules (e.g., CD4, CD28, CTLA-4, ICOS) on the cell surface leads to cell aggregation and reduces their migration ability. Physical restrictions not only induce alterations in the cell microenvironment, but also affect cellular nutrition and metabolism, which results in 3D spheroid structure formation. Besides, in GBFs for simulation of microgravity such as clinostat and RPM, cancer cells are affected by the shear forces [16], which also might lead to the formation of spheroids.

Previous studies have demonstrated that cytoskeletal proteins are the preferred target upon exposure to real or simulated microgravity [26, 28, 71, 72]. It is observed that the cytoskeletal structures undergo several changes besides structural rearrangement: (1) shortened and disordered; (2) obvious changes in IF content, showing ring aggregation around the nucleus; (3) loosely arranged keratin network with ‘alveolar' structure; (4) increased vimentin content and number of positive cells; (5) decreased number of MF and a tendency to aggregate the cytoplasm at the edge of cells. MT, MF, and keratin are associated with cell movement, which could reduce cell migration and increase the chances of cell aggregation and sphere formation under the constraints of adhesion molecules, ECM and physical restrictions. Due to the circular aggregation of the IF around the nucleus, destruction of nuclear function might lead to barriers in transcription and translation and activate the apoptotic pathway, resulting in the reduced cell viability and 3D spheroid formation. In addition, such a change in the IF could also cause the cells to become more rounded, resulting in the cells coming into contact with each other and not being easily separated, thereby forming a sphere.

Real and simulated microgravity induces 3D growth in many cells [73, 74] as a result of neutralization of sedimentation. SMG on the RPM alters the physiological function of cell, such as the cytoskeleton, gene expression and ECM, all of which can promote 3D growth [54, 75].* VEGFA* and* VEGFD* were both significantly upregulated under SMG using a RPM [30]. Both of genes showed coinstantaneous upregulation when observed in human ovarian carcinomas [76]. *α* and *β* actin are key cytoskeletal factors, which are downregulated by* VEGF*. Downregulation of *α* and *β* actins increases the ability of cells adhering to each other and forming a sphere with 3D growth. Upregulation of* VEGFA* indicates neoangiogenesis, low-differentiation and progression in cancer cells, whereas* VEGFD* plays a key role in neovascularization and formation of lymphatic vessels in cancer tissues [77, 78]. Both of them can mimic the microenvironment of tumor growth* in vitro*, indicating that* VEGFA* and* VEGFD *can affect 3D growth. Plasminogen activator inhibitor-1 (PAI-1) was down-regulated upon* VEGF* upregulation. Plasminogen accumulation inhibits sphere formation. Therefore, the inhibitory effect of plasminogen activator is weakened due to the down-regulation of PAI-1 and upregulation of* VEGF*, thus contributing to sphere formation.

The cytoskeleton transports mechanical signals to cells, and thus affects both biochemical pathways [79, 80] and gene expression [81, 82].  Table 4 shows that some studies have examined changes in the expression of genes, predominantly those involved in apoptosis, cytoskeleton, ECM, proliferation, migration and the cell cycle [31, 35, 48, 50, 51]. The effect of real and simulated microgravity on genes was evaluated in FTC-133 human follicular thyroid cancer cells on the RPM, 2D clinostat, Shenzhou-8 Space mission and TEXUS 52 sounding rocket flight mission [31, 33–35, 48]. These genes can be grouped into the following categories: transcription factors—*AKNA*,* E2F2*,* IRX3*, and* SOX9*; factors related to cell adhesion—*PCDHβ5*,* PCDH7*,* EPAC1*,* ITGB8*, and* ITGA3*; factors involved in angiogenesis—tumor necrosis factor receptor superfamily member 12A (*TNFRSF12A*),* CAV1*,* PRKCA*,* VEGFA*, and* IL8*; factors related to cytoskeleton organization—actin-binding LIM protein 1 (*ABLIM1*),* KRT7*,* KRT80*,* TUBB2B*, and* TUBG1*; factors involved in apoptosis—*CAV1*,* PRKCA*,* BIRC3*,* BIRC5*,* BIRC7*,* BCL2*,* BCL3*, TNFRSF12A, and Bcl-2-like protein 12 (*BCL2L12*); factors related to ECM organization—*SERPINH1*,* ITGB8*; factors involved in cell proliferation—*FOSL1*,* TGFBR3*,* ANXA3*; and factors related to cell cycle—*CCND2*,* CCNE2*,* CCNE1*, and* CCNA1*. These genes are associated with all aspects of the cell.* PRKCA* and* CAV1* are involved in angiogenesis and apoptosis. On one hand, by promoting angiogenesis to increase the invasion of tumor cells, these genes promote distant metastasis; on the other hand, apoptosis is promoted and the physiological activity of cells is weakened through the up-regulation of these genes. In addition, at the molecular level, SMG affects the expression of various genes in cells, resulting in phenomena such as spheroid formation, increased apoptosis, reduced migration, and cell cycle arrest, but the intrinsic link between these genes still remains to be discovered.

To date, the detailed reasons for 3D spheroid formation are still unknown. Well-known mechanisms of 3D spheroid formation on thyroid cancer cells were presented by Albi and collaborators under real and simulated microgravity, such as changes of gene expression for extracellular matrix and cytoskeleton proteins, thyrocyte phenotype, and the expression and movement of cancer molecules from thyrocytes to colloids [5]. Based on the evidence available, we can infer that the 3D growth patterns [73], GBFs for simulation of microgravity [16], cytoskeletal rearrangement [26, 38, 83, 84], gene alteration [31, 35, 48], and apoptosis [35, 85] might also participate in the 3D spheroid structure formation. In general, these factors interact and intertwine together to promote the formation of 3D spheroids under real and simulated microgravity.

### 3.2. Alteration of the Cytoskeleton under Microgravity

A common result in most cells under real and simulated microgravity is the change in cytoskeletal components: actin, MF, and MT [26, 29, 33]. Riwaldt and collaborators [30] performed an experiment to examine MT organization* in vitro* and hypothesized that simulated microgravity affects the self-organization of MT networks; they observed that some shorter, no preferential MTs existed in UCLA RO82-W-1 follicular thyroid cancer cells after 24 h of RPM. Vassy and collaborators [37] also observed the same result in MCF-7 breast cancer cells after 48 h of spaceflight. Their studies proposed that MT networks are gravity-dependent reaction–diffusion processes, which are characterized by dynamic growth and shortening of MTs. Thus, SMG can drive this process to produce shorter, no preferential MTs. MTs provide architectural support for cellular organization, shape, motility, and replication. It is tempting to speculate that the disorders caused by exposure to SMG have the potential to substantially affect cell metastatic capacity, migration, adhesion and invasion. Furthermore, microtubule associated protein (MAP) is attached to MT polymers and participates in the assembly of MTs and increases the stability of MTs. Dysfunction of MAP in different cells affects both MT and MF networks, which also leads to the phenomenon mentioned above.

The IF content ranged differently in the total cell protein and is the main component of the cell system structure [86, 87]. Thus, IFs play an important role in nuclear morphology, migration, distribution and signal transduction [88–98]. IFs consist of a variety of proteins, among which cytokeratin and vimentin are the core proteins. Normally, the cytokeratin network surrounding the nucleus and substrate exhibits an immobilization model whereas the meshes are regular in size, formerly described as “alveolar” [38, 99]. MCF-7 breast cancer and ML-1 thyroid cancer cells subjected to real microgravity exhibited a loosely organized perinuclear cytokeratin network in conjunction with prolonged time spent in mitosis, compared to NG control [26, 38]. Similar results in MCF-7 breast cancer cells were also shown by J. Vassy et al. [37, 38], which were both studied in real microgravity. The cell is a relatively closed environment, where any change in one of its components can affect local tension. The endoplasmic reticulum, Golgi apparatus and nucleus as some cell components could be attacked by caspases [87, 99], resulting in the destruction of cytokeratin and local tension. Consequently, local tension or forces could be used to explain the loose cytokeratin network. These findings were consistent with the basic prediction of cell tension [100, 101]. In ML-1 and ONCO-DG1 thyroid follicular carcinoma cells, vimentin content was increased and exhibited an irregular arrangement on the RPM and clinostat [25, 36]. Impaired function (membrane potential reduction) plays an important role in some cell physiological activities because thyroid cancer cells are rich in mitochondria. Under specific interaction between a subdomain of vimentin's non-helical domain and mitochondria, the binding of the mitochondria to vimentin can significantly increase its membrane potential. Most functions of mitochondria depend on the membrane potential produced and maintained by its intima. The increased content and structural rearrangement of vimentin can inhibit binding to mitochondria. Decrease in membrane potential is accompanied by the release of apoptotic factors in the membrane space, which leads to activation of caspases and apoptosis. In MDA-MB-231 breast cancer cells, the structural disorder of vimentin networks occurs in the form of compact aggregation closely related to the nucleus on the RPM [41]. Vimentin has nucleophilic properties and shows loss of polarity in microgravity and aggregation of nucleated-vimentin polymers; besides, the IFs of vimentin can move towards the nucleus, suggesting that it may be immobilized on the nuclear membrane.

In ML-1 thyroid cancer, MCF-7 and MDA-MB-231 breast cancer cells under real and simulated microgravity, actin stress fibers were decreased and showed disappearance of complex cytoplasmic networks mainly distributed at the edge of the cells [29, 38, 41]. The same result was observed by Vassy et al. and Kopp et al. [37, 75]. Stress fibers are composed of a large number of parallelly arranged actin, myosin, promyosin, and *α*-comyosin; MF is skeletal fibers with an actin diameter of about 7 nm, also known as actin fibers. The MF, its binding protein, and myosin form a chemomechanical system that can produce mechanical motion and have the function of contraction in the cytoplasm. Stress fibers are in a state of equal length contraction (increased tension without length shortening) in cells, which is important for cell movement, protein synthesis, and gene expression. Due to decreased quantity and abnormal distribution of stress fibers in SMG, the stress fibers change from isometric shrinkage to isotensional contraction or show a mixture of the two and have a profound effect on several activities, such as increasing apoptosis and 3D spheroid formation. Suitable assembly of MT and filamentous actin can be influenced by real microgravity according to previous space studies [33, 102]. Actin monomers can form actin polymers under the action of ATP, and cell movement is related to the polymer. ATP is affected by microgravity, which hinders the formation polymer and changes in MF structure. Association of proteins is also involved in the polymer formation, which is one of the possible reasons for the change in MF structure. The movement of cancer cells in the matrix comprises four cyclic reciprocating steps: (1) formation and extension of the head pseudopod; (2) establishment of new attachment sites; (3) contraction of the cells; and (4) retraction of the tail. Rho GTP enzymes, especially RhoA, Rac1, and Cdc42 play an important role in the activation of focal adhesion kinase (FAK), regulation and migration of cellular morphology, phosphorylation of the myosin light chain, and enhancement of cell contraction [103–105]. SMG significantly reduces the activation of FAK and Rho family proteins (e.g., RhoA, Rac1 and Cdc42), resulting in decreased tumor migration and 3D spheroid formation.

## 4. Extracellular Proteins

The ECM was increased in ML-1, ONCO-DG1 thyroid cancer cells and endothelial cells under real and simulated microgravity [5, 20, 25, 27, 32, 36, 39, 46, 52]. Increased ECM and a large amount of Cav1, which is associated with several proteins such as PKC or KDR in the cell membrane, can lead to firm anchoring of the cells in ECM and inhibit 3D spheroid formation [30, 32, 106]. This appears to contradict the conclusion that microgravity promotes the formation of 3D spheroids. However, the formation of 3D spheres is affected by several factors such as cytoskeletal arrangement, gene alteration, apoptosis, and the ECM. Therefore, we can infer that 3D sphere formation is a complex regulatory process, and a single factor can not change the whole trend. ECM is an important tissue barrier to prevent tumor metastasis. Matrix metalloproteinases (MMPs) can degrade all kinds of protein in ECM, destroy the histological barrier of tumor cell invasion, and play a key role in tumor invasion and metastasis [107]. In general, decrease in MMPs at the gene and protein level in microgravity is beneficial to inhibit cell proliferation, cell migration, and invasion, which are all important processes of tumor metastasis [22, 24, 38, 42]. In addition, MMP-2 and MMP-9 are aggressive pseudopods, which contribute to the degradation of ECM* in vivo* and* in vitro*. MMP-2 and MMP-9 may be involved in the regulation of angiogenesis in tumor invasion and metastasis and may promote the release and migration of vascular endothelial cells from basement membrane (BM) by degradation of BM and ECM. MMP-2 can upregulate angiogenic cytokines through the following processes: (1) degradation of collagen IV, which exposes the binding sites of integrin *α* V *β* 3 and promotes the migration of endothelial cells [108]; (2) promoting the synthesis of VEGF and activating the inactive TGF-*β*; (3) degradation of matrix components, release of basic FGF and VEGF combined with matrix components [109]; and (4) release of membrane-bound TNF-*α* and other cytokines that promote angiogenesis by the degradation of vascular BM. MMPs may also have adverse effects on tumor angiogenesis. It has been found that MT1-MMP regulates endothelial factor exfoliation and inhibits tumor angiogenesis [110]. It is speculated that the role of MMPs in tumor angiogenesis is dual. When exposed to real and simulated microgravity, the microenvironment required for tumor cell growth changes dramatically, and the role of MMPs in promoting angiogenesis may dominate. TIMP inhibits and inactivates MMPs by non-covalent binding with MMPs, which protects the ECM from degradation and remodeling by MMPs. Increasing the expression of TIMP by inhibiting MMPs, and the enzymes and pathways related to its mechanism may downregulate MMPs and reduce their activity, thereby inhibiting the invasion and metastasis of tumor cells. SMG using a 3D clinostat system increases the expression of TIMP genes [22], resulting in MMP down-regulation, which can reduce ECM degradation and decreased invasiveness. These results are consistent with the later description of tumor cell proliferation and migration ability under microgravity.

## 5. Cell Cycle

It is widely recognized that exposure to real and simulated microgravity leads to impressive modification in the cell cycle [23, 111].  Table 2 lists the cell cycle changes in some tumor cell lines under real and simulated microgravity. In MCF-7 breast cancer cells, although G_2_/M or G_2_+M was increased, the time that starts to increase is not the same. The earliest increase in G_2_/M can be seen after 24 h of SMG followed by 72 h. Similarly, after 24 h and 48 h under SMG, an increase in the G_2_+M phase is observed. For the MDA-MBB-231 cell line, the G_2_/M began to increase after 72 h of exposure, which shows no temporal consistency with the MCF-7 cell line. For the G_0_/G_1_ or G_0_+G_1_ phase, contrasting results were observed: G_0_+G_1_ increased in the SGC-7901 human gastric cancer cells after 48 and 72 h of SMG exposure, but G_0_/G_1_ decreased in H460 lung cancer cells after 24 h. It was thus observed that the cell cycle changes were not exactly the same. There are differences on rotating devices aiming to simulate microgravity (e.g. clinostat system and RPM) that might account for the alteration in cell cycle. Compared to the RPM, the amount of shear forces in clinostat is lower [16]. This shows that higher shear forces are acting on the cancer cells during the RPM [16] might lead to different results. From the point of cell characteristic, this is because both of cells have their unique characteristics, which ultimately lead to differences in the cell cycle; furthermore, microgravity – at least SMG – might have a greater effect on different phenotypes of the same cell line, which magnifies some of the occult features of normal gravity.

*Cyclin* has a significant effect on tumor progression and metastasis by inducing cell proliferation or apoptosis. Cyclin dependent kinase (CDK) plays an important role in G_1_/S and G_2_/M transition, which can affect the cell cycle progress. In a previous study, it was observed that the mRNA levels of CDK_1_, CDK_2_, CDK_4_, CCNB1 and CCNE1 in DLD-1 colorectal cancer cells were decreased under SMG using a RCCS-HARV and RPM [21, 43]. The levels of cyclin D1, which is a protein encoded by* CCND1* and is necessary to regulate the cell cycle from G_1_ to S and promote cell differentiation and apoptosis [112, 113], decreased in H460 lung cancer cells under SMG using a RPM [23], MDA-MB-231 and MCF-7 [40, 41] breast cancer cells using a RPM and RCCS-HARV, leading to cell aggregation in the S and G_2_/M phase. Cyclin D1 could not bind and activate CDK4 specific to the G_1_ phase at present, which blocked the phosphorylation of the G_1_ cycle inhibitor protein (Rb), resulting in the dissociation of unphosphorylated RB protein from the E2F transcription factor. Thus, upon G_1_ blockage, the S phase was reduced, and G_2_/M was increased. Increase in S phase may be due to short exposure to SMG with no change in cyclinD1 and CDK4, resulting in no cell cycle arrest. Synthesis of CDK-cyclin protein complexes, which phosphorylate specific target proteins and promote cell cycle development, is hindered as a result of the decreasing CDK. SMG affects CyclinA-CDK1 and CyclinB-CDK1 complexes and blocks cell transformation in G_2_/M, resulting in increased G_2_/M or G_2_+M. The CyclinE-CDK2 complex is not formed due to the CDKs disability of phosphorylation/dephosphorylation, resulting in cell cycle arrest at G_1_/S under SMG.* DOC-1R* specifically binds to CDK2, inhibits the nuclear transport of CDK2 protein, thus preventing CDK2 protein from forming a complex with cyclin, and also results in a G_1_/S block.

The 14-3-3 protein, which is important for such vital regulatory processes as mitogenic signal transduction and cell cycle control, was evaluated in MCF-7 cells subjected to SMG [43, 114]. 14-3-3 protein can form a positive feedback loop by directly increasing the transcriptional activity of P53 and inhibiting progress to G_1_/S. In mammalian cells, activation of CDC2 is a necessary step to enter the M phase [115], whereas CDC25C dephosphorylation activates CDC2, thus enabling cells to enter the M phase [116]. Therefore, activation of CDC25C is necessary to promote the cell cycle process [117]. Under SMG, CHK1 is activated because of DNA damage, which phosphorylates the Ser216 site of CDC25C and binds 14-3-3 protein; CDC25C is thus trapped in the cytoplasm, resulting in cell cycle arrest at G_2_ phase [118]. 14-3-3 protein binds and negatively regulates the CDC25 phosphatase. CDC25A is a central modulator that enters the S phase, and dephosphorylates and activates CDK2, whereas the 14-3-3 protein binds to CDC25A and forms a cytoplasmic block, resulting in S blockage. Vimentin has been shown to interact with the regulatory 14-3-3 protein [119–121]. During the S/G_2_/M phase of the cell cycle, 14-3-3 protein binds to phosphorylated keratin 18 (K18), which strongly reduces the interaction between 14-3-3 and other proteins. Vimentin and keratin 17 (K17) can also bind to 14-3-3 protein; thus phosphorylated keratin and vimentin can bind 14-3-3 protein during the cell cycle process, affecting the interaction of 14-3-3 with other molecules, such as CDC2 or CDC25C, which in turn leads to cell cycle arrest.

## 6. Proliferation

Table 3 describes the alteration of cell proliferation under real and simulated microgravity. Proliferation capacity is found to be reduced in different cancer cells as a result of the reduced expression of proliferation markers using a clinostat system (e.g., IGFBP-2, PCNA, MKI67) [24, 44, 45]. In addition, cycle arrest and low expression of TGF-R, TGF-a, and TGF-*β* also lead to the reduction in proliferation [19].

IGFBP-2, PCNA, and MKI67 are necessary proteins for cell growth and the cell cycle. The function of PCNA-CDK-cyclin protein complexes is decreased due to the low expression of PCNA under SMG, resulting in phosphorylation blockage and G1 arrest. P21, a strong CDK inhibitor, can completely inhibit almost all the activity of CDK/cyclin complexes and play an important role in many cell pathways. SMG induces the expression of P21 protein, which in turn inhibits cell cycle and DNA replication. The PCNA-P21 complex can form tetramers with multiple CDK/cyclin complexes, which inhibits CDK activity, thus affecting the phosphorylation of RB protein and the release of transcription factors that bind to it, resulting in cells failing to pass the G1 phase check point. The effects of IGFBP-2 and MKI67 on proliferation under SMG might include inhibition of microtubule assembly, thus hindering the process of spindle pulling sister chromatid separation and moving toward the cell poles, which keeps the spindle assembly test points active and then hinders the cell cycle progress from the middle to the later stage, and cell cycle arrest at the mitotic stage (M phase). This allows cyclin B1 and CDC2 protein kinase to remain active, resulting in Bcl-2 phosphorylation and activation of c-Jun amino terminal kinase, which induces a reduction proliferation [122].

SMG can reduce the activity of the Cyclin B/Cdc2 complex by decreasing the level of cell cycle dependent kinase CDK7 protein, downregulating the Thr^161^ phosphorylation of Cdc2 protein mediated by CDK7, increasing the phosphorylation of Cdc2 protein Thr^161^/Tyr^15^, and upregulating the expression of P53, P21, and P27 proteins, resulting in G_2_/M arrest [123]. CyclinB1, as a member of the cell cycle family, is a key factor to control cell entry into the G_2_/M detection point. Overexpression of Cyclin B1 accelerates G_2_ phase cells into the M phase, resulting in excessive cell proliferation. SMG inhibits the expression of Cyclin B1 protein and blocks the formation of heterodimer Cyclin B1/CDK1, which also results in G_2_/M phase arrest. Under these factors, cell proliferation is inhibited because of G_2_/M phase arrest, which prevents the cell from completing mitosis.

TGF-*β* regulates cell proliferation through its receptors. For example, TGF-*β*1 activates Smad2/3 through a heteropolymer (two sets of TGFBR2/TGFBR1 dimer) composed of TGFBR2/TGFBR1, which activates the Smad-dependent signaling pathway. Smad signaling can inhibit the expression of c-myc and promote the expression of cell cycle inhibitory proteins P21, P15, and so on, which leads to stagnation of the cell cycle. This pathway results in inhibition of cell proliferation by TGF-*β*1 [124, 125]. Besides, TGFBR2 / TGFBR1, TGFBR2 or TGFBR1 can activate non-Smad signaling pathways, such as MAPK, Rho-like GTPases, and PI3K. Among these pathways, MAPK and PI3K can promote cell proliferation [126–128]. However, only a decrease in cell proliferation was observed under SMG, so it was speculated that Smad signaling plays a major role in cell proliferation. EGF can bind epidermal growth factor receptor (EGF-R) protein with tyrosine kinase activity, activate the downstream PI3K/AKT signaling pathway, and maintain normal cell growth, proliferation, and development [129, 130]. SMG reduces the expression of EGF-R, causing AKT phosphorylation in the signaling pathway and thus decreasing cell proliferation.

## 7. Apoptosis

Table 3 shows most tumor cell lines (thyroid carcinoma cells ML-1 and ONCO-DG1, gastric carcinoma cells SGC-7901, U251 glioma cells, MDA-MB-231 breast cancer cells and BL6-10 melanoma cells) under SMG present the phenomenon of increased apoptosis [25, 36, 41, 44, 45, 47], but decreased apoptosis in MIP-101 poorly differentiated colorectal carcinoma ceils [19] and absence of obvious apoptosis in squamous cell carcinoma of the cervix (SCC) and ML-1 thyroid cancer are also observed [26, 109]. Cell cycle arrest, abnormal expression of apoptosis-related proteins (increased P21, P53, Fas and Bax; decreased the Bcl-2), tumor suppressor genes (*PTEN* and* P53* up-regulation) is often found in cancer cell lines [20, 25, 36, 41, 43, 45] (MDA-MB-231 and MCF-7 breast cancer cells, ML-1 and ONCO-DG1 thyroid carcinoma cells, DLD-1 colorectal cancer cells and U251 glioma cells) with increased apoptosis. Cell cycle arrest is affected by many factors, of which* PTEN* and* P53* are important. Upregulation of* PTEN* significantly increases the number of G_1_ phase cells and significantly reduces the number of S phase cells. The S phase is a key stage of DNA synthesis, which inhibits the malignant proliferation of cancer cells.* P53* induces cell cycle arrest by regulating the transcription of* P21*,* 14-3-3σ*,* GADD45*, and* Cyclin B1*, then monitoring the checkpoint of the G_1_ and (or) G_2_/M phase. Moreover, P53 transcription activates apoptotic genes such as* Bax*,* Noxa*,* Puma*, and* Perp*, and induces apoptosis. Apoptosis is an active process that involves the activation, expression and regulation of a series of proteins. Among them, Caspase family proteins, Bcl-2 family proteins and P53 proteins are the most involved in the signal transduction of apoptosis. Caspase-8 triggers the cascade reaction to activate Caspase-3 under the action of the apoptotic signal, and then activation of the activated caspase lysis specific substrate leads to apoptosis. Caspase activation depends on the cleavage or self-activation of other caspase at its aspartic acid site. Cells enter apoptosis under SMG through activation of Caspase-3 via endogenous or exogenous routes. Direct degradation of intracellular structural and functional proteins by Caspase-3 can induce apoptosis. Bax allows some ions and small molecules such as cytochrome C (CYC), to pass through the mitochondrial membrane and enter the cytoplasm, thus causing apoptosis; in contrast, Bcl-2 can block the activity of Bax to form pores and prevent some small molecules from permeating freely, thus protecting cells from apoptosis. In a SMG environment, the apoptosis-promoting protein Bax was increased, while the anti-apoptotic protein Bcl-2 was decreased, and thus the ratio of the two proteins, resulting in morphological changes related to apoptosis, such as chromatin condensation, membrane blebbing, loss of nuclear envelope, and cellular fragmentation into apoptotic bodies. P53 responds to SMG as a transcription factor and induces the expression of downstream proteins such as P21, Mdm2 and Bax, which regulate the cell cycle and apoptosis. The mechanism of P53 mediated apoptosis may be as follows: (1) the mitochondrial membrane loses integrity during apoptosis, and CYC is released into the cytoplasm, leading to activation of caspase fragmentation. The anti-apoptotic protein Bcl-2 and pro-apoptotic protein Bax regulate CYC release. (2) Reactive oxygen species (ROS) are potent activators of mitochondrial damage and apoptosis. Many proteins increase the Ros production, so oxidative stress can be induced by P53. P53 can up-regulate Fas and induce Fas-mediated death. The tumor suppressors* PTEN* and* FOXO3* were upregulated under SMG using a RCCS in DLD1 colorectal cancer cells [20]; on one hand,* PTEN* inhibited the transformation of PIP2 to PIP3 and inhibited the activation of AKT, whereas on the other hand, upregulation of the CDK inhibitor CDKN2D and CDKN2B induced downregulation of AKT and apoptosis. It is believed that the decrease in the S and G_2_/M phase and cell death were closely associated with G_1_ phase arrest. These results suggest that cell death under simulated microgravity is through activation of cell cycle inhibition pathways.

It is observed that after SMG exposure, the time of apoptosis began to increase and was not the same. Apoptosis of ML-1 and ONCO-DG1 thyroid cancer cells was increased after 24 h exposure of RPM and 3D clinostat [25, 36], whereas MDA-MB-231 breast cancer and SGC-7901 gastric adenocarcinoma cells showed increased apoptosis after 72 h exposure of RPM and a rotating clinostat respectively [41, 44]. The possible reason might be that different methods for simulation have been used, including 3D clinostat and RPM. Furthermore, we speculate that, in addition to apoptosis related proteins, autophagy is also an important factor affecting this differential expression. Autophagy has the function of inducing cell death by promoting apoptosis. During the early stage of cancer progression, autophagy in ML-1 and ONCO-DG1 thyroid cancer cells showed a significantly increased trend compared to other cell lines, resulting in a marked advance in the apoptosis time of these two cell lines. miRNA is a small molecule which can regulate gene expression and plays an important role in autophagy of tumor cells. After exposure to SMG, the expression of miRNAs was increased resulting in increased autophagy. The specific mechanism underlying this observation remains to be found.

Survivin, involved in cell division and apoptosis suppression, is an inhibitor of apoptosis proteins and is influenced by SMG [41, 131, 132]. Although the exact mechanism of Survivin function needs further studies, evidences have exhibited that Survivin may inhibit apoptosis in two main ways: first, by directly interacting with effector Caspase-3 and Caspase-7 [124], and preventing their activation and interfering with the caspase-independent AIF pathway of cell death [133]; second, by interacting with cyclin kinase CDK4, p34cdc2 and promoting apoptosis signal transduction. This is the reason why apoptosis is reduced under SMG.

The demonstration that the different rotation devices achieved in SMG is associated with a decrease in proliferation and increase in apoptosis suggests that even relatively low levels of shear stress are associated with changes in the expression of molecules that regulate cell proliferation and apoptosis. However, further work is necessary to demonstrate whether this postulate is correct.

## 8. Cell Migration

Cell migration is a multi-step coordinated process that can be reflected by a wound healing scratch test. In MCF-7 breast cancer [42] and A549 lung adenocarcinoma [24] cells, the wound healing rate was decreased after 24 h of clinostat whereas in DLD-1 colorectal cancer and A549 lung adenocarcinoma cells, it was increased after 12 and 24 h of RCCS and 3D clinostat, respectively [20, 22]. As can be seen from these results, migration ability between cell lines is not the same, which is consistent with previous studies [21, 30, 40–42]. Even in the case of human lung adenocarcinoma A549 cells, significantly different results were observed. One study [22] showed that SMG increases the migration ability after 24 h of 3-D clinostat, whereas other [24] studies showed the opposite. The dynamic changes in MF and MT provide the main driving force for cell movement. Although it is the same cell line, migration capacity is affected by the differential expression in MF and MT as a result of the appearance of cell phenotypes with different functions and physiological characteristics. In addition, the cell growth environment, treatment level, exposure factors, results analysis and statistical methods also can affect the results. The different principles of the simulation devices (e.g. clinostat system and RCCS) may also account for the alteration in cell migration. These need to be studied in future experiments.

It has been demonstrated that vimentin is necessary for wound healing both in cultured cells and in animal models [125, 126] and is associated with invasive behavior in prostate and breast cancer [128, 129, 134]. Epithelial mesenchymal transition (EMT) is an important event in the process of cancer metastasis. Vimentin is an important marker of EMT and a necessary regulatory factor for the migration of mesenchymal cells, whose activity is interrelated with E-cadherin expression. Previous studies have shown that decreased function of E-cadherin could result in aggressiveness, de-differentiation, and metastasis in many carcinomas [135–137]. The available evidence suggests that SMG can rearrange vimentin and form nucleo-vimentin polymers. This alteration can reduce the number of vimentin molecules that exert physiological effects, leading to altered migration ability of cancer cells. Furthermore, EMT is also involved in other steps in the cancer process. Under SMG, the TGF-*β* signal pathway simultaneously inhibits the expression of two EMT-induced genes* snail1* and* snail2* and promotes apoptosis.

Actin is the main protein controlling cell movement. Inspired by the research of Block et al. [138] who found cell contraction under real microgravity and SMG, it is speculated that the rearrangement of actin under SMG shortens the MFs, which might lead to obstruction of cell tail contraction and affect migration. There is a functional correlation between the microtubule network and the microfilament network, which interact with each other to control the process of cell migration. Furthermore, it is also postulated that the destruction of the microtubule network under SMG can affect the formation of pseudopodia and hinder the movement of cells. Cells turn on their own internal switch and initiate the migration process under SMG. Cdc42 is known as an important switch through the continuous activation and inactivation of GEF and GAP. However, the intracellular and extracellular signal transduction disturbance due to SMG and the decrease in Cdc42 enzyme activity lead to corresponding changes in migration.

## 9. Conclusion

Compared with normal gravity, tumor cells change under altered gravity conditions, as shown in SMG and in some cases in real microgravity. These changes include cell aggregation, cytoskeleton rearrangement, cell cycle arrest, migration and apoptosis. The 3D growth pattern, GBFs for simulation of microgravity, cytoskeletal rearrangement, gene alteration and apoptosis might also participate in the 3D spheroid structure formation. Recently, a large number of GBFs designed by different concepts have been constructed to SMG on the ground. It is known that the one-axis clinostat and two-axis RPM modes are subjected to the shear forces. However, compared to the one-axis clinostat, the amount of shear forces in two-axis RPM modes are higher. These shear forces have a strong influence on function in cancer cells. This shows that higher shear forces are acting on the cancer cells during the RPM, which might lead to the different results on the two-axis RPM and one-axis clinostat.

Spaceflight missions are very rare and costly. Therefore, the development of GBFs like the RPM or clinostat gave new ideas in study of microgravity. The direction of the gravity vector in these GBFs has undergone a constant (2-D clinostat) or random (RPM) change, with the gravity level averaged to near zero along with rotation and time. Therefore, they appeared suitable for studying on earth and can be used for exploring the alterations in cancer cells under SMG. Over the last several years, many experiments have shown that exposure to SMG alters biological processes. We hope to find the effects of SMG on cancer cells biological functions using the GBFs, to further study the mechanism using this model and to deeply understand the growth behavior and function of human cancer cells. However, for exploring cancer in microgravity, it is destined to be a winding road.

## Figures and Tables

**Table 1:** Publications (1997-2018) describing 3D spheroid formation and the alteration of morphology and function of human cancer cells under real and simulated microgravity.

Samples(cell line)	Exposure Devices	Speed (RPM^1^)	Exposure time	Major result	Ref.
Colorectal cancer					

MIP-101	r-*μ*g / Two Spaceflights	—	10 d and 12 d	Proliferation and apoptosis decreased	[19]
DLD1,HCT116,SW620	s-*μ*g / RCCS-HARV	10	48 h	Cell clumping and apoptosis increased	[20]
DLD1	s-*μ*g / RCCS-HARV	27	72 h	Cell cycle arrested	[21]

Lung cancer					

A549,H1703	s-*μ*g / 3D clinostat system	5	36 h	Proliferation rates decreased in H1703; while migration ability increased in both	[22]
H460	s-*μ*g / RPM^2^	—	48 h	Apoptosis increased	[23]
A549	s-*μ*g / MG-3 clinostat system	30	72 h	Metastatic potential,proliferation,while migration and invasion reduced	[24]

Throid cancer					

ML-1	s-*μ*g / RPM^2^	10	72 h	IF, ECM and apoptosis increased	[25]
ML-1	r-*μ*g / PFC	—	22 s	F-actin/cytokeratin cytoskeleton altered, no signs of apoptosis	[26]
ML-1	s-*μ*g / RPM^2^	—	72 h	3D spheroid formation, ECM increased	[27]
ML-1	s-*μ*g / RPM^2^	10	7 d	3D spheroid formation	[28]
ML-1	s-*μ*g / RPM^2^	10	11 d	actin and tubulin altered	[29]
UCLA RO82-W-1	s-*μ*g / RPM^2^	10	24 h	3D spheroid formation, ECM increased	[30]
UCLA RO82-W-1	s-*μ*g / RPM^2^	10	7 d	3D spheroid formation	[28]
FTC-133	r-*μ*g / Shenzhou-8 space mission	—	10 d	3D spheroid formation	[31]
FTC-133	r-*μ*g / International Space Station	—	12 d	Enhanced production of proteins related to the ECM	[32]
FTC-133	r-*μ*g / TEXUS-52	—	369 s	Significant alterations of the cytoskeleton	[33]
FTC-133	s-*μ*g / 2D clinostat	60	3 d	3D spheroid formation, CAV1 and CTGF decreased in spheroids	[34]
FTC-133	s-*μ*g / RPM^2^	10	3 d	3D spheroid formation, CAV1 and CTGF decreased in spheroids	[34]
FTC-133	s-*μ*g / RPM^2^	10	24 h	3D spheroid formation, apoptosis increased, CTGF decreased in spheroids	[35]
ONCO-DG1	s-*μ*g / Clinorotation	10	24 h	Cell clumping and apoptosis increased	[36]

Breast cancer					

MCF-7	r-*μ*g / Spaceflight	—	48 h	Cytokeratin network were looser, cell cycle arrested and proliferation decreased	[37]
MCF-7	r-*μ*g / Spaceflight	—	48 h	Cytoskeleton changes, cell cycle prolonged	[38]
MCF-7	s-*μ*g / RPM^2^	12.5	24 h	3D spheroid formation, apoptosis-related proteins increased	[39]
MDA-MB-231,MCF-7	s-*μ*g / RCCS	20-25	5 d	Cyclin D1 decreased	[40]
MDA-MB-231	s-*μ*g / RPM^2^	10	72 h	G_2_/M inibited and cyclin D1 decreased	[41]
MCF-7	s-*μ*g / MG-6C clinostat system	30	72 h	Cell invasion and migration decreased,	[42]
MCF-7	s-*μ*g / RPM^2^	—	48 h	Proliferation decreased, while apoptosis-related proteins increased	[43]

Other cancer					

SGC-7901 gastric cancer	s-*μ*g / MG-3 clinostat system	30	72 h	Proliferation and apoptosis decreased	[44]
U251 glioma	s-*μ*g / 2D clinostat system	—	72 h	Proliferation and apoptosis decreased	[45]
Multiple human tumor	s-*μ*g / RCCS-HARV	25	7 d	Spheroid formation	[46]

Table 1. d, day; h, hour; s, second; RPM^1^, rounds per minute; PFC, parabolic flight campaign; r-*μ*g, real microgravity; s-*μ*g, simulated microgravity; RCCS-HARV: Rotational Cell Culture System-High Aspect Ratio Vessel; RCCS: Rotational Cell Culture System; RPM^2^: Random Positioning Machine.

**Table 2:** Publications (1997-2018) describing cell cycle alterations in human cancer cells under simulated microgravity.

Samples (cell line)	Exposure Devices	Speed (RPM^1^)	Exposure time	Result	Ref.
Breast cancer					

MDA-MB-231,MCF-7	s-*μ*g / RCCS	20-25	5 d	S phase and G_2_/M phase increased	[40]
MCF-7	s-*μ*g / RPM^2^	—	48 h	slightly accumulate in G_2_/M	[43]
MDA-MB-231	s-*μ*g / RPM^2^	10	72 h	S phase increased after 24 h; while after 72 h S phase decline,	[41]
				G_2_/M phase increased	
MCF-7	s-*μ*g / MG-6C clinostat system	30	72 h	G_2_+M phase increased after 24 and 48 h	[42]

Other tumor					

SGC-7901 gastric cancer	s-*μ*g / MG-3 clinostat system	30	72 h	G_0_+G_1_ Phases increased significantly, while the S+G_2_+M phase	[44]
				decreased at 48, 72 h	
H460 lung adenocarcinoma	s-*μ*g / RPM^2^	—	48 h	S phase increased, while G_0_/G_1_ phase decreased	[23]

Table 2. d, day; h, hour; RPM^1^, rounds per minute; s-*μ*g, simulated microgravity; RCCS: Rotational Cell Culture System; RPM^2^: Random Positioning Machine.

**Table 3:** Publications (1997-2018) describing proliferation and apoptosis in human cancer cells under real and simulated microgravity.

Samples (cell line)	Exposure Devices	Speed (RPM^1^)	Exposure time	Result	Reason	Ref.
Breast cancer						

MDA-MB-231	s-*μ*g / RPM^2^	10	72 h	Apoptosis increased after 72 h	Bax protein increased,while Bcl-2 decreased	[41]
MCF-7	s-*μ*g / RPM^2^	—	48 h	No obvious apoptosis	No obvious change in apoptosis related	[43]
					protein,such as P53, Bax, Bcl-2, P27	
MCF-7	s-*μ*g / MG-6C rotating clinostat system	30	72 h	Proliferation decreased	Partial remodeling of G_2_/M	[42]
MCF-7	s-*μ*g / RPM^2^	12.5	24 h	Apoptosis increased after 24 h	Fas, Casp8, Bax and p53 increased	[39]

Thyroid cancer						

ML-1	s-*μ*g / RPM^2^	10	72 h	Apoptosis increased after 24 h	The protein of P53,Fas and Bax increased,	[25]
					while Bcl-2 decreased	
ONCO-DG1	s-*μ*g / 3D clinostat system	10	24 h	Apoptosis increased after 24 h	Bax increased and Caspase-3 activated,whlie Bcl-2	[36]
					decreased	

Colorectal cancer						

DLD-1	s-*μ*g / RCCS-HARV	10	48 h	Proliferation reduction	Tumor suppressor PTEN and FOXO3 upregulated	[20]
MIP-101	r-*μ*g / STS-70 and STS-85 space shuttle flights	—	10 d and 12 d	Proliferation and apoptosis reduced	The low expression of EGF-R,TGF-a,and TGF-*β*	[19]

Other tumor						

U251 glioma	s-*μ*g / 2D clinostat system	—	72 h	Proliferation decreased, while apoptosis increased	P21 upregulated, while IGFBP-2 downregulated	[45]
					
SGC-7901 gastric cancera	s-*μ*g / MG-3 clinostat system	30	72 h	Proliferation decreased after 48 h and 72 h, while apoptosis increased after 72 h	PCNA decreased	[44]
					
H460 lung adenocarcinoma	s-*μ*g / MG-3 clinostat system	30	72 h	Proliferation decreased	MKI67 decreased	[24]
BL6-10 melanoma cells	s-*μ*g / RPM^2^	—	24 h	Apoptosis increased after 24 h	Suppressing Uev1A/TICAM/TRAF/NF-kB-* *-Regulated anti-apoptosis	[47]

Table 3. d, day; h, hour; RPM^1^, rounds per minute. r-*μ*g, real microgravity; s-*μ*g, simulated microgravity; RCCS-HARV: Rotational Cell Culture System-High Aspect Ratio Vessel; RPM^2^: Random Positioning Machine.

**Table 4:** The basic experimental conditions of human cancer cells involved genes changes under real or simulated microgravity.

Samples(cell line)	Exposure Devices	Exposure time	Ref.
FTC-133 thyroid cancer cells	s-*μ*g/RPM	24 h	[35]
FTC-133 thyroid cancer cells	r-*μ*g / Shenzhou 8 space mission and s-*μ*g / RPM	10 d	[48]
FTC-133 thyroid cancer cells	r-*μ*g / Shenzhou 10 space mission and s-*μ*g / RPM	10 d	[31]
MCF-7 breast cancer cells	s-*μ*g/RPM	5 d	[50]
L-540 and HDLM-2 Hodgkin's lymphoma cancer cells	s-*μ*g / 3D Clinostat	3 d	[51]

Table 4. d, day; RPM, rounds per minute. r-*μ*g, real microgravity; s-*μ*g, simulated microgravity; RPM: Random Positioning Machine.

## Abbreviations

RPM: Random positioning machine
RWV: Rotating wall vessel
ECM: Extracellular matrix
MT: Microtubule
MF: Microfilament
IF: Intermediate filament
NG: Normal gravity
2D: Two-dimensional
3D: Three-dimensional
*μ*g: Microgravity
SMG: Simulated microgravity
EGFR: Epidermal growth factor receptor
TGF: Transforming growth factor
TGF-R/a/*β*: Transforming growth factor R/a/*β*
GBFs: Ground-based facilities
NASA: National Aeronautics and Space Administration
LSMMG: Low-shear modeled microgravity
RCCSS: Rotating cell culture systems
HARVs: High-aspect rotating vessels
PKC: Protein kinase C
MAPKs: Mitogen-activated protein kinases
RAF-1: Rapidly accelerated fibrosarcoma-1
HIF-1: Hypoxia-inducible factor-1
CD4/28: Cluster of differentiation 4/28
CTLA-4: Cytotoxic T lymphocyte-associated antigen-4
ICOS: Inducible costimulatory
VEGFA/D: Vascular endothelial growth factor A/D
VEGF: Vascular endothelial growth factor
PAI-1: Plasminogen activator inhibitor-1
AKNA: AT-hook transcriptional factor
E2F2: E2F transcription factor 2
IRX3: Iroquois homeobox 3
SOX9: SRY-related HMG-box gene 9
PCDH*β*5/7: Protocadherin Beta 5/7
EPAC1 (RAPGEF3): Rap guanine nucleotide exchange factor 3
ITGB8: Integrin beta8
ITGA3: Integrin *α*3
TNFRSF12A: Tumor necrosis factor receptor superfamily member 12A
CAV1: Caveolin-1
PRKCA: Protein kinase C *α*
IL8: Interleukin 8
ABLIM1: Actin-binding LIM protein 1
KRT7/80: Keratin 7/80
TUBB2B: Tubulin beta 2B
TUBG1: Tubulin gamma 1
BIRC3/5/7: Baculoviral IAP repeat-containing 3/5/7
BCL2/3: B-cell lymphoma 2/3
TNFRSF12A: TNF receptor superfamily member 12A
BCL2L12: Bcl-2-like protein 12
SERPINH1: Serpin family H member 1
FOSL1: FOS like 1
TGFBR1/2/3: Transforming growth factor beta receptor 1/2/3
ANXA3: Annexin A3
CCND2: Cyclin D2
CCNE1/2: Cyclin E1/2
CCNA1: Cyclin A1
CCNB1: Cyclin B1
MAP: Microtubule associated protein
Rho A: Ras homolog A
RAC1: Rac family small GTPase 1
CDC42: Cell division cycle 42
CDC2: Cell division cycle 2
CDC25C: Cell division cycle 25C
CDC25A: Cell division cycle 25A
CDC25: Cell division cycle 25
CDKN2D/2B: Cyclin dependent kinase inhibitor 2D/2B
FAK: Focal adhesion kinase
KDR: Kinase insert domain receptor
MMPs: Matrix metallo proteinases
MMP-2/9: Matrix metallo proteinase 2/9
BM: Basement membrane
FGF: Fibroblast growth factor
TNF-*α*: Tumor necrosis factor *α*
TIMP: Tissue inhibitor of metalloproteinase
CDK: Cyclin dependent kinase
P34CDC2 (CDK1): Cyclin dependent kinase 1
CDK4/7: Cyclin dependent kinase 4/7
DOC-1R: Deleted in oral cancer-1 related
CHK1: Checkpoint kinase 1
Ser216: Phospho-CDC25C
K17: Keratin 17
IGFBP2: Insulin like growth factor binding protein 2
PCNA: Proliferating cell nuclear antigen
MKI67: Marker of proliferation Ki-67
Smad2/3: Drosophila mothers against decapentaplegic protein 2/3
PIK3: Phosphatidylinositol-4,5-bisphosphate 3-kinase
AKT: Protein kinase B
PTEN: Phosphatase and tensin
GADD45: Growth arrest and DNA damage-inducible 45
PUMA: P53-upregulated modulator of apoptosis
PERP: P53 apoptosis effector related to PMP-22
CYC: Cytochrome C
ROS: Reactive oxygen species
FOXO3: Forkhead box O3
EMT: Epithelial mesenchymal transition
GEF: Guanine nucleotide exchange factor
GAP: Growth associated protein.
